# Effectiveness of common antidepressants: a post market release study

**DOI:** 10.1016/j.eclinm.2021.101171

**Published:** 2021-10-25

**Authors:** Farrokh Alemi, Hua Min, Melanie Yousefi, Laura K Becker, Christopher A Hane, Vijay S Nori, Janusz Wojtusiak

**Affiliations:** a Department of Health Administration and Policy, George Mason University, Fairfax, VA; b OptumLabs; c School of Nursing, College of Health, George Mason University; d OptumLabs Visiting Fellow

**Keywords:** antidepressants, major depression, Effectiveness, comorbidities, medical history, post-market release

## Abstract

**Background:**

This study summarizes the experiences of patients, who have multiple comorbidities, with 15 mono-treated antidepressants.

**Methods:**

This is a retrospective, observational, matched case control study. The cohort was organized using claims data available through OptumLabs for depressed patients treated with antidepressants between January 1, 2001 and December 31, 2018. The cohort included patients from all states within United States of America. The analysis focused on 3,678,082 patients with major depression who had 10,221,145 antidepressant treatments. Using the robust, and large predictors of remission, and propensity to prescribe an antidepressant, the study created 16,770 subgroups of patients. The study reports the remission rate for the antidepressants within the subgroups. The overall impact of antidepressant on remission was calculated as the common odds ratio across the strata.

**Findings:**

The study accurately modelled clinicians’ prescription patterns (cross-validated Area under the Receiver Operating Curve, AROC, of 82.0%, varied from 77% to 90%) and patients’ remission (cross-validated AROC of 72.0%, varied from 69.5% to 78%). In different strata, contrary to published randomized studies, remission rates differed significantly and antidepressants were not equally effective. For example, in age and gender subgroups, the best antidepressant had an average remission rate of 50.78%, 1.5 times higher than the average antidepressant (30.30% remission rate) and 20 times higher than the worst antidepressant. The Breslow-Day chi-square test for homogeneity showed that across strata a homogenous common odds-ratio did not exist (alpha<0.0001). Therefore, the choice of the optimal antidepressant depended on the strata defined by the patient's medical history.

**Interpretation:**

Study findings may not be appropriate for specific patients. To help clinicians assess the transferability of study findings to specific patient, the web site http://hi.gmu.edu/ad assesses the patient's medical history, finds similar cases in our data, and recommends an antidepressant based on the experience of remission in our data. Patients can share this site's recommendations with their clinicians, who can then assess the appropriateness of the recommendations.

**Funding:**

This project was funded by the Robert Wood Johnson foundation grant #76786. The development of related web site was supported by grant 247-02-20 from Virginia's Commonwealth Health Research Board.

**Research in context**

**Evidence before this study**

The majority (60%) of depressed patients do not benefit from their first antidepressant. To help clinicians improve their prescription patterns, a number of attempts have been made including: (a) development of consensus-based guidelines, genetic profiling, and clinical decision aids, usually based on a predictive model. These efforts have not led to an effective decision aid for prescribing antidepressants.

**Added value of this study**

The current study improved accuracy of clinical decision aids by examining effectiveness of antidepressants after statistically removing observed confounding/selection bias in the data. The study examined 11,472,471 antidepressant treatment episodes. To remove bias, the study identified features of patients’ medical history that either predicted remission or affected prescription of antidepressants. These features were used to partition the data into 16,770 distinct subgroups. This study summarizes experience of remission for cases that fell within each subgroup.

**Implications of all the available evidence**

An optimal antidepressant is recommended based on which subgroup matches patients’ medical history. The study provides a free decision aid (see http://hi.gmu.edu/ad) to match patients to subgroups and recommend a course of action.

Alt-text: Unlabelled box

## 1 Introduction

Antidepressants are one of the most frequent medications taken in the U.S.; 11% of the U.S. population takes antidepressants [1,2]; and yearly sales of antidepressants exceed several billion dollars [1]. For some time, it has been known that the majority (60%) of depressed patients do not benefit from their first antidepressant [3,4]. To help clinicians improve their prescription patterns, several consensus-based guidelines have been published [[2], [3], [4], [5], [6], [7], [8], [9], [10]]. Even with the availability of the published guidelines, prescribing the right antidepressants remains a hit and miss effort. Others have tried to improve selection of antidepressants through genetic profiling, but reviews show that these efforts have not been very fruitful [11]. A number of investigators have tried to improve precision of antidepressant prescriptions through predictive modeling [[12], [13], [14], [15], [16], [17], [18], [19], [20], [21], [22]].

The current study adds to available literature on predictive modeling by reporting effectiveness of antidepressants, after removing observed confounding/selection bias in the data. The confounding/bias occurs when clinicians prescribe certain antidepressants (e.g. second tier antidepressants) more often than other (e.g. first tier antidepressants) to treatment-resistant or severely-depressed patients. The antidepressants prescribed for more severe depression will be less likely to be effective primarily because of the patient's characteristics, as opposed to anything inherently due to the medication itself. The current study rigorously controls for observed confounding/selection bias, a step not done in previous predictive models. In addition, previous studies [[23], [24], [25], [26], [27]] have relied on selective set of symptoms or measures, calculated typically at baseline of other research studies. Unlike these previous attempts, we rely on the entire medical history of the patient, including all comorbidities, and all previous medications of the patient.

## 2 Methods

**Study Design:** This is a retrospective, observational, matched case control study of depressed patients treated with antidepressants. Cases were patients who took a specific antidepressant and controls took other antidepressants. The study examined the performance of one antidepressant against others. The study does not determine if the patient should take antidepressants. Instead, for patients whose clinician has prescribed, or will prescribe, an antidepressant, the study clarifies which one has the highest evidence of remission. The analysis was done per treatment episode and not per patient as patients in our data tried different antidepressants.

**Source of Data:** The cohort was organized using administrative claims data available through OptumLabs [28]. The data included 71,721,417 patients during the timeframe between January 1, 2001 and December 31, 2018. Among these, 11,472,471 took one or more antidepressants and 6,897,748 also had a diagnosis of major depression. We excluded 2,790,721 patients who had a short medical history (defined as being eligible for coverage for at least 1 year and 100 days). After all inclusions and exclusions, we focused the analysis on 3,678,082 unique patients in 10,221,145 treatment episodes. The average follow-up period was 2.93 years, after their first antidepressant use. This cohort included a total of 15,096,055 person-years of data.

**Outcome**: In clinical studies of effectiveness of antidepressants, the main outcome of interest is patient-reported remission of depression symptoms. Since this outcome is often missing in claims data, investigators have devised proxy measures for remission [29-[30], [31], [32], [33], [34]]. We relied on patterns of antidepressant use to indicate remission, in particular we relied on: (1) duration of use, (2) reaching a therapeutic dose, (3) switch from one antidepressant to another, and (4) augmentation of current antidepressant. An index based on these factors accurately predict (Cross-validated Area under Receiver Operating Curve = 0.93) the probability of remission [35]. This index assumes the patient is in remission if all four conditions are present, meaning that duration exceeded 10 weeks, the dose exceeded therapeutic level, and there was no switch or augmentation of the medication in the first 10 weeks. In addition, we relied on remission reported by clinicians within their codified diagnoses (e.g. ICD 9 code 296.25 indicates “partial or unspecified remission.”). When the clinician had indicated remission, then we assumed remission independent of the pattern of use of the medication.

There are multiple reasons for stopping a medication (switching to another medication, or having a short intake of medication, or not receiving minimum effective dose). Some patients may stop taking the medication because it is not effective, others may stop because of side-effects. The present study does not clarify why a medication was stopped, just that it was stopped.

**Treatment:** This study separately analyzed the effectiveness of monotherapy with 15 most common antidepressant during the study period, including: Amitriptyline, Bupropion, Citalopram, Desvenlafaxine, Doxepin, Duloxetine, Escitalopram, Fluoxetine, Mirtazapine, Nortriptyline, Paroxetine, Ropinirole, Sertraline, Trazodone, and Venlafaxine. Monotherapy with less common antidepressants besides the 15 studied, and combination of any two of the 15 antidepressants studied, when filled on the same day, were classified as treated with “Other” medications.

**Covariates:** The current study statistically controlled for how patients’ history of illness, procedures, or medications affected response to antidepressants. The analysis included every diagnosis, as a separate binary variable. This resulted in 15,356 inpatient and 16,811 outpatient predictors derived from the patients’ diagnoses. For example, we statistically controlled for cognitive disorders [22], substance use disorders [36,37], obesity [38], insomnia [39], cerebrovascular diseases [40,41], hormone imbalances [42,43], cancer [44], or post-traumatic stress disorder [45], and other diseases.

In addition, we defined a separate variable for each mental health procedure. Procedures were identified using either Current Procedural Terminology or ICD codes. A total of 4,364 binary procedure variables for mental health encounters were included in the analysis.

We also included 4,253 medications as generic drug names, measured through the pharmacy claims data. Finally, we also created measures using the number of previous treatment episodes and remission status in the last treatment episode.

In total, we had 40,784 unique binary predictors in the year prior to the start date of the antidepressant. The analysis focused on the 1,000 variables using the SAFE rule for excluding variables before the analysis [46]. Even though 1,000 predictors may seem large, proportional to the size the data it was not, as we had a total of 360,930,690 records on 10,171,970 antidepressant episodes of 3,657,828 unique individuals.

**Missing Variables:** The outcome variable, i.e. patient reported symptom remission, was missing in all cases and was imputed from a surrogate measure constructed from patterns of antidepressant use. When covariates were missing, it was assumed the patient did not have the diagnosis or the medication.

**Methods of Analysis:** In this observational study, we statistically control for observed confounding and selection bias. The study focuses on confounding by indication. Confounding by indication means that a comorbidity, procedure, or medication (all measured in the year prior to taking the antidepressant) was associated with selection of the antidepressant and/or the remission outcome. Observed confounding in the data is usually removed through propensity scoring [47]. More recent theoretical progress has shown that stratification can also do the same [48,49]. To be effective, stratification should occur for all factors that affect either remission or propensity for prescribing the medication. In particular, we followed these steps:

(1) *Identification of Predictors of Prescriptions:* As recommended by Shojaie and colleagues, we used Least Absolute Shrinkage and Selection Operator (LASSO) logistic regression to predict chances of prescribing a medication [50]. In these regressions, the response variable was 1, if the antidepressant was selected, and 0 otherwise. The independent variables were the 1,000 variables selected from medical history. LASSO regression is preferred to ordinary regression because of the need to reduce the number of features that should be stratified. Separate LASSO regressions were done for each antidepressant. We used 5-fold cross-validation with the one standard error rule to reduce the number of variables in the LASSO model. These procedures led to less than 1% loss in predictive accuracy of the model.
(2) *Identification of Predictors of Remission (Parents in Markov Blanket of Remission):* LASSO logistic regression was used to see how medical history affected remission rates. For each antidepressant, first the data were limited to the patients who received the antidepressant. In these LASSO regressions, the response variable was remission, 1 if the patient had experienced remission and 0 otherwise. The regression parameters were set as described in step 2. The independent variables were 1,000 medical history features.
(3) *Check of Robustness:* In identification of predictors of either remission or propensity of prescribing an antidepressant, bootstrapping was used to examine if variables selected in the LASSO regressions were robust. LASSO models were trained on 40 datasets created by sampling with replacement from the original training data. A final logistic regression model was fitted on the variables which had the same non-zero sign in 95% of the 40 models.  Age and gender were always included in the list of robust variables to make interpretation of findings easier.
(4) *Creation and Trimming of Strata:* A stratum (also referred to as subgroup of patients) was constructed from combination of robust predictors. Because of the number of robust variables, many combinations occurred only once. To have sufficient data within each stratum, less important variables were dropped until at least 99 cases fell within each stratum. Excluding age and gender, the importance of variables was measured by the absolute value of the coefficient in the final LASSO regressions.

The above analytic steps were carried out using Python software, version 3.8.2, and Python libraries: numpy 1.18.2, scikit-learn 0.23.1, and h2o 3.28.1.2. Python code for constructing Causal Networks through robust LASSO regressions is available at https://lasso-bbn.readthedocs.io.

The overall impact of antidepressant on remission, the effect size across strata, was calculated as the common odds ratio [51]. Even though a common odd can always be calculated, it is not a reasonable statistic, if the impact of antidepressant on remission is not homogenous. The Breslow-Day chi-square test for homogeneity was used to test if, across strata, a homogenous common odds-ratio existed [52]. When a common odds ratio does not exist, then effectiveness of antidepressants should be examined separately within each stratum.

**Sensitivity Analysis**: Regression coefficients are often sensitive to data sample. The study increased the robustness of these coefficients through repeated regressions in 40 random sampling of the data.

**Compliance with STROBE Criteria:** This manuscript is compliant with STrengthening the Reporting of OBservational studies in Epidemiology (STROBE). A checklist is provided as an online supplement.

**Role of Funding Source:** This project was funded by the Robert Wood Johnson foundation and by Virginia's Commonwealth Health Research Board. Neither funding organizations, nor OptumLabs, had any role in preparation, or approval, of the content of this paper.

## 3 Results

This cohort included patients from all states within United States of America. The mean age of the cohort was 46.54 years (standard deviation of 17.48); 16.92% were over 65, 6.5% were teenagers (see Table 1 for details). In this cohort, due to privacy regulations, race was assigned based on the racial distribution in the individual's county of residence. County-based race information was available for 99.83% of individuals. The county of residence for the majority of the patients were predominantly white (77.24%), 14.01% Black and 13.56% Hispanic.

**Table 1:** Demographic Distribution of Patients in the Cohort (Race was inferred from county where the patient resided)

	N = 3,678,082 Unique Patients
**Age in Years**	mean: 46.54; std: 17.48 median: 46.0
13-19 years	252,086 (6.85%)
20-40 years	1,157,601 (31.47%)
41-64 years	1,654,834 (44.75%)
65-79 years	499,249 (13.57%)
80+ years	123,312 (3.35%)
**Gender**	
Female	2,551,031 (69.36%)
Male	1,127,051 (30.64%)
**Insurance**	
Commercial	3,003,628 (81.66%)
Medicare Advantage	673,045 (18.30%)
Missing	1,409 (0.04%)
**Available County Information**	3,672,128 (99.84%)
**County Level Race**	3,672,008 (99.83%)
White	mean: 77.24; std: 14.33
Black	mean: 14.01; std: 12.29
Asian	mean: 4.40; std: 4.84
Hispanic	mean: 13.56; std: 13.87
Hawaiian	mean: 0.12; std: 0.96
Native American	mean: 1.44; std: 2.04
Other	mean: 5.50; std: 4.96
**Follow-up Years**	mean: 2.93; std: 2.72; median: 1.98 IQR: 0.95-3.97

The number of robust variables for predicting propensity of prescribing antidepressants ranged from 51 to 206 predictors, with more prevalent medications having higher number of predictors (see Table 2). The Area under the Receiver Operating Curves (AROC) on the 10% holdout test dataset ranged from 77.2%, for Venlafaxine, to 90.5%, for Escitalopram. The average AROC for predicting propensity of medications was 82%. 15 models were constructed to predict remission for 15 different antidepressants. The average prevalence of remission ranged from 3.1% to 49.3%.  The cross-validated AROC ranged from 69.2% to 78.5%, with an average of 72.0%.  In these analyses, the number of robust predictors ranged from 22, for Nortriptyline, to 195, for Sertraline, and 232 for Other category of antidepressants.

**Table 2:** Cross-validated Accuracy of LASSO Logistic Models

Antidepressant	Prescribing the Antidepressant			Remission from Antidepressant		
	Prevalence	AROC	# Predictors	Prevalence	AROC	# Predictors
Amitriptyline	2.8%	85.0%	134	3.1%	77.7%	40
Bupropion	8.9%	77.9%	156	6.1%	74.0%	34
Citalopram	9.1%	81.0%	168	48.7%	69.6%	173
Desvenlafaxine	0.8%	82.3%	83	47.8%	74.6%	58
Doxepin	0.5%	84.1%	51	22.2%	72.8%	38
Duloxetine	4.4%	79.7%	118	39.1%	69.2%	129
Escitalopram	10.6%	79.0%	155	20.5%	70.4%	108
Fluoxetine	8.7%	82.9%	95	45.5%	70.6%	151
Mirtazapine	1.8%	84.6%	124	16.8%	69.8%	44
Nortriptyline	0.9%	84.0%	62	9.7%	72.3%	22
Other	24.0%	77.4%	206	35.1%	72.6%	232
Paroxetine	4.7%	84.9%	136	43.7%	69.9%	123
Ropinirole	0.6%	90.5%	55	33.9%	68.5%	47
Sertraline	12.3%	81.9%	122	49.3%	70.5%	195
Trazodone	4.6%	79.3%	105	3.0%	78.5%	24
Venlafaxine	5.3%	77.2%	72	48.9%	71.0%	138

AROC is Area under the Receiver Operating Curve. Separate models were conducted for each medication. Other category included combination of listed medications and medications not listed.

To reduce observed confounding in calculation of impact of antidepressants on remission, we stratified robust predictors of remission/antidepressant prescription. Each antidepressant was analyzed separately. Between 80 to 271 variables were used to create the strata for different antidepressants. A total of 38,811 to 1,043,206 strata were initially created. In strata with too few observations, the less important variables were dropped until trimmed-strata had at least 100 observations. There were 16,770 trimmed strata. Patients can be matched to the stratum that shares most of their features; thus, because of how the strata was constructed, every patient falls into only one stratum.

Within each trimmed stratum, the effect size is reported as the proportion of the patients who experienced remission. It may be helpful to examine some of these strata in more detail. Table 3 shows remission rates for 5 most common antidepressant for female teenagers, in 30 of the 16,770 strata. The full set of strata are available in the online supplement 3. For all female teenagers with no other features (i.e. ignoring any predictors of remission), Citalopram had the best remission rate. Citalopram was also the preferred antidepressant for female teenagers when (a) they were not responsive to Escitalopram, (b) had a history of taking Citalopram, or (c) had recurrent major depression. In contrast, Sertraline was preferred for female teenagers diagnosed with (a) malaise and fatigue, (b) unspecified anxiety, (c) unspecified insomnia, (d) reporting routine infant or child health check, (e) headache, (f) abnormal weight gain, (g) myopia, (h) or history of taking Ethinyl Estradiol/Drospirenone. Female teenagers had higher remission rates on Fluoxetine when the patient had a history of (a) routine gynecological exam, (b) was not responsive to Sertraline, (c) or had previously taken Fluoxetine. In summary, different antidepressants were recommended for female teenagers who had no relevant medical history (a common situation) and teenagers who did have a variety of other factors in their medical history. The former group is typically included in the randomized clinical trials and published widely. The latter group is not typically included in randomized trials, as patients with specific comorbidities are often excluded. The optimal antidepressants in the group typically enrolled in randomized trials varies from those typically excluded. This study is unique in providing information on effectiveness of antidepressants for patients with multiple comorbidity.

**Table 3:** Remission Rates of Antidepressants in Subgroups of Female Teenagers

Strata for Female Teenagers	Bupropion	Citalopram	Escitalopram	Fluoxetine	Sertraline
Ignoring predictors of remission		**43.15%**	11.78%	34.07%	41.43%
Other Malaise & Fatigue	5.21%		13.56%	38.74%	**52.59%**
Anxiety State, Unspecified	4.72%		11.93%	31.07%	**50.93%**
History of use of Escitalopram Oxalate | Last Antidepressant Different & No Remission	3.00%	**47.62%**		33.45%	37.37%
History of use of Citalopram Hydrobromide	4.75%	**34.02%**	13.93%	23.39%	27.27%
History of use of Escitalopram Oxalate	3.53%	**40.04%**	18.21%	22.46%	25.18%
Insomnia, Unspecified				36.15%	**43.36%**
History of use of Amitriptyline HCL	6.42%	27.03%	10.87%	23.49%	**27.66%**
Routine Infant or Child Health Check		32.92%		31.46%	**38.11%**
Headache	3.74%			31.90%	**41.03%**
History of use of Sertraline HCL	3.81%	24.03%	12.76%	24.31%	**34.13%**
Depressive Disorder, Not Elsewhere Classified	2.72%	33.49%	13.31%	31.68%	**41.80%**
Abnormal Weight Gain	3.57%				**41.99%**
History of use of Bupropion HCL | Last Antidepressant Different & No Remission			15.54%		**42.73%**
Routine Gynecological Examination			15.02%	**38.95%**	
History of use of Citalopram Hydrobromide | Last Antidepressant Different & No Remission	2.83%		15.68%	34.13%	**35.78%**
History of use of Amitriptyline HCL | History of use of Fluoxetine HCL				**37.00%**	
Last Antidepressant Different & No Remission | History of use of Sertraline HCL	2.96%	23.71%	10.89%	**33.42%**	
Major Depressive Disorder, Recurrent Episode, Unspecified	37.03%	**62.14%**	41.62%	44.22%	50.71%
Last Antidepressant Different & No Remission| History of use of Paroxetine HCL	4.88%		11.57%		**39.66%**
Myopia	5.68%	35.60%	15.61%	40.44%	**40.33%**
History of use of Fluoxetine HCL	5.86%	23.68%	11.50%	**30.42%**	24.91%
History of use of Citalopram Hydrobromide | Psychotherapy, 45 Minutes with Patient		**43.45%**		15.57%	14.56%
History of use of Amitriptyline HCL | History of use of Sertraline HCL					**36.39%**
History of use of Dextroamphetamine/Amphetamine			9.04%	28.70%	**43.03%**
Adjustment Disorder with Mixed Anxiety & Depressed Mood	2.42%		13.18%		**36.21%**
History of use of Fluoxetine HCL | Last Antidepressant Different & No Remission	3.49%	33.61%	11.55%		**35.37%**
Routine General Medical Examination at Health Care Facility			10.53%		**42.99%**
Last Antidepressant Different & No Remission | History of use of Sertraline HCL	12.68%		27.45%	**43.75%**	
History of use of Ethinyl Estradiol/Drospirenone		34.29%	13.45%		**43.72%**

Cell entries are remission rates for the antidepressant for a female teenager in the strata. Bold entries show antidepressants with highest remission rate. The complete data for all age, gender, and medical history strata in the online supplement.

To examine if there is a common odds ratio of remission across the strata, we relied on Breslow Day Chi-square test of homogeneity. Table 4 shows the resulting statistics. For all antidepressants, the test of homogeneity of the odds ratios across the strata was rejected (p value < 0.0001). While common odds ratios can be calculated, and is reported in Table 3, there is too much variability across the strata for these odds to be meaningful. Since the common odds ratio does not exist, then the best course of action is to match the patient to the strata; and use the odds of remission for the matched stratum.

**Table 4:** Effectiveness of Antidepressant across Strata

Antidepressant	Number of Significant Predictors	Number of Strata	Number of Trimmed Strata	Breslow Day Statistic for Homogeneity	Common Odds Ratio (99% Confidence Interval)*
Amitriptyline	148	227,526	749	10,362 (p-value<0.0001)	1.25 (1.23 to 1.27)
Bupropion	163	662,577	2,371	193,397 (p-value<0.0001)	1.14 (1.13 to 1.15)
Citalopram	263	759,321	2,258	122,764 (p-value<0.0001)	1.03 (1.03 to 1.03)
Desvenlafaxine	116	64,650	239	14,275 (p-value<0.0001)	0.99 (0.98 to 1.01)
Doxepin	73	36,783	148	4,570 (p-value<0.0001)	1.16 (1.14 to 1.18)
Duloxetine	194	390,553	1,253	46,622 (p-value<0.0001)	1.06 (1.05 to 1.06)
Escitalopram	202	848,032	2,765	107,639 (p-value<0.0001)	1.07 (1.07 to 1.07)
Fluoxetine	187	669,013	2,237	149,309 (p-value<0.0001)	1.01 (1.01 to 1.02)
Mirtazapine	142	153,949	436	10,785 (p-value<0.0001)	1.09 (1.08 to 1.1)
Nortriptyline	74	62,961	254	5,015 (p-value<0.0001)	1.16 (1.14 to 1.18)
Paroxetine	204	390,144	1,230	55,010 (p-value<0.0001)	1.06 (1.05 to 1.06)
Ropinirole	76	51,933	184	4,168 (p-value<0.0001)	1.16 (1.15 to 1.18)
Sertraline	252	1,028,431	3,162	219,717 (p-value<0.0001)	1.02 (1.01 to 1.02)
Trazodone	113	331,735	1,258	81,815 (p-value<0.0001)	1.23 (1.22 to 1.25)
Venlafaxine	164	434,208	1,485	84,394 (p-value<0.0001)	1.00 (1 to 1.01)
Other	321	2,156,363	6,804	452,940 (p-value<0.0001)	1.02 (1.01 to 1.02)

⁎ No Common Odds Ratio Existed across Strata

Table 5 reports the performance of select antidepressants in age and gender subgroups (ignoring all other aspect of the medical history), a small subset of the strata in this study. Clinicians prescribed a variety of antidepressants within these strata. Table 5 provides the antidepressant with highest response rate in bold. In these subgroups, the best antidepressant was on average 20.45 times more effective than the worst antidepressants. The average remission rate was 30.30%. If all clinicians had prescribed the antidepressant with the best rate, then 50.78% of patients would have been in remission. If so, then 1.5 times more patients, or 1,608,914 more episodes, would have had symptom remission.

**Table 5:** Remission Rates for Select Antidepressants in Age & Gender Subgroups

Age & Gender Groups	Bupropion	Citalopram	Desvenlafaxine	Escitalopram	Fluoxetine	Sertraline	Venlafaxine
Age: 13-19 Female	6.68% (19,891)	28.39% (25,292)	28.25% (1,586)	13.75% (40,046)	26.25% (50,572)	29.85% (58,857)	29.9% (11,264)
Age: 13-19 Male	5.29% (16,243)	25.05% (12,475)	26.45% (726)	13.92% (19,697)	24.72% (25,261)	26.88% (30,690)	26.29% (5,572)
Age: 20-40 Female	5.59% (191,664)	42.94% (169,331)	43.98% (17,933)	18.09% (220,077)	40.6% (200,708)	45.5% (277,958)	45.31% (104,843)
Age: 20-40 Male	5.84% (79,494)	41.38% (66,637)	41.37% (6263)	18.8% (88,852)	39.1% (54,677)	42.21% (92,840)	42.62% (38,943)
Age: 41-64 Female	6.35% (305,046)	52.12% (278,067)	51.51% (30,090)	22.84% (315,428)	50.24% (294,013)	54.33% (342,158)	51.05% (200,900)
Age: 41-64 Male	6.97% (134,314)	50.78% (114,603)	46.87% (9,766)	22.83% (131,628)	49.66% (85,883)	52.27% (139,402)	49.89% (64,838)
Age: 65-79 Female	6.36% (44,203)	55.11% (97,030)	52.92% (3,987)	22.58% (84,940)	58.61% (59,520)	58.43% (109,590)	54.6% (37,979)
Age: 65-79 Male	6.71% (22,908)	55.66% (38,491)	49.74% (1,512)	22.45% (34,205)	57.3% (21,185)	58.1% (45,521)	54.04% (14,118)
Age: 80-89 Female	6.12% (4,872)	44.28% (26,382)	54.68% (406)	16.63% (23,604)	48.91% (9,073)	49.02% (32,855)	48.57% (6053)
Age: 80-89 Male	5.57% (2,387)	46.57% (9,659)	54.07% (135)	17.27% (8,611)	48.73% (3,587)	51.45% (12,973)	52.34% (2052)

Cell values show percent of patients experiencing remission. Number of treatment episodes receiving the antidepressant is given in parentheses. Best remission rates are in bold. Within the age and gender group, bold antidepressants are significantly (alpha<0.05) better than non-bold ones. Full report of monotherapy with 15 antidepressants is available in online supplement.

## 4 Discussion

This study showed that both clinicians’ prescription patterns and patients experience of remission can be modelled accurately. For the clinician's prescription of antidepressants, the cross-validated AROC was 0.82, a relatively high level of prediction accuracy. For predicting symptom remission, the cross-validated AROC was 0.72, a moderate level of accuracy. These data suggest that medical history can anticipate both how clinicians prescribe and which patients will experience remission.

In the literature, numerous articles have reported that antidepressants have, on average, similar impact on symptom remission [53-[54], [55], [56]]. Our data, and the fact that patients/clinicians continue to search for better antidepressants, suggests otherwise. Remission rates changed by 20-fold in different age and gender subgroups of patients. No medication was always best. For individual patients, there is clearly a right and wrong antidepressant, depending on which one of the 16,770 subgroups match the patient's medical history.

Data suggest that there is room for improving clinicians’ prescription patterns. If all clinicians had prescribed the best antidepressant, then 1.5 times more episodes could have ended with remission. This means that out of 9,199,617 episodes, 3,063,210 experienced remission; but if all patients had received the best medication and experienced the same remission rates as the study data, then an additional 1,608,914 episodes would have had symptom remission. The study suggests that millions of patients could benefit from a decision aid that can help them select the right medication. Improving prescription patterns will help large number of patients.

These findings should be considered in context of several limitations. First, this is a convenient, and not random, sample. Generalization from the experiences of these patients to others in United States may not be reasonable. At the same time, this sample of more than 9 million treatment episodes was large enough to provide stable estimates of patient experiences in small subgroups. Furthermore, we undertook rigorous statistical adjustments to remove confounding in observational data. These steps suggest that study findings may generalize to other patients in United States.

Second, we did not have access to patient reported symptom remission. Although a previous study had shown that 95% of variation in the patient reported remission was accurately predicted by our surrogate measure [57]; this single study has not been replicated by others. Additional research should be done on whether pattern of use of antidepressants can serve as a surrogate measure of patient-reported remission.

Third, this analysis did not include all antidepressants. The analysis did not include less common older antidepressants. It did not include more recent newer antidepressants. We also did not examine the remission rate associated with combination of antidepressants. We focused on mono-therapy by the 15 most common antidepressants.

Finally, fourth, the analysis involved multiple comparisons and no multiplicity corrections were made. Typically, when there are multiple comparisons, an adjustment is made by requiring a stricter significance threshold, so as to compensate for the number of inferences being made. Given that we are reporting p-values less than 0.0001, multiplicity adjustment may not be necessary. At this p value, in 16,770 comparisons, we would be wrong in 2 occasions.

We constructed 16,770 subgroups based on large, robust, predictors of remission and propensity to select an antidepressant. These subgroups may not correspond to current ways clinicians classify who should receive various antidepressants. For example, these subgroups do not correspond to measures of severity of depression. Some of the strata are also quite unexpected: for instance, there is a stratum with female teenagers with myopia. Myopia was a robust and large predictor of remission and therefore the basis for constructing the strata. At the same time, there may be no theoretical or biological reason to assume myopia affects depression symptom remission. Although, recent studies show that some SSRI antidepressants may cause dry eye; and therefore, may not be suitable for patients with myopia [58]. In thousands of subgroups of patients that we examined, some combination of factors may inevitably make sense statistically but not clinically. The question, of course, is what should change – should we have created the subgroups based on current theories or alternatively, as we did, based on predictors of remission. The advantage of relying on statistical predictors is that it captures the largest variation in the remission outcome. It reduces the possibility of erroneously attributing remissions to the treatment as opposed to the patient's medical history. The disadvantage is that it may not fit current clinical theories or practices.

This study provides two ways for identifying optimal antidepressant for a patient: (a) one could use the 15 robust regression models to predict from the patient's medical history the probability of remission for each antidepressants; or (b) one could match patient's medical history to the 16,770 strata and select the antidepressant with best remission rate in the stratum. Both approaches are reasonable, and likely to lead to the same conclusions; but we prefer to use the strata because many question the relevance of population level models to individual-level decisions. This study divided patients experiences into thousands of subgroups. Even though the optimal antidepressant for each subgroup is in the online supplement to this paper, it is difficult to review so many subgroups. To assist, we have organized a free web site at http://hi.gmu.edu/ad (also see http://MeAgainMeds.com), where patients can provide their medical history and find the subgroup that matches their history. Within that subgroup, they can see the experiences of others and select the antidepressant with the highest remission rate. They can, then, take the site's recommended medication to their clinicians for further discussion and possible prescription.

This study, while suggestive, is not conclusive. Additional studies are needed. Our findings may not generalize to other situations. A randomized prospective clinical trial is needed to see if clinicians/patients who follow the advice of this study will, in fact, experience better remission than clinicians/patients who do not.

## Contributors

FA organized the study, obtained the funding, supervised the analysis, and development of the decision aid. HM and MY provided clinical input; LKB organized the data; and CAH and VSN conducted the analysis and provided statistical advice. JW prepared the decision aid. CAH, VSN, and LB had access to the raw data. All authors participated in writing the paper.

## Funding

This project was funded by the Robert Wood Johnson foundation grant #76786. The development of related web site was supported by grant 247-02-20 from Virginia's Commonwealth Health Research Board.

## Data Sharing Statement

Investigators who seek access to the study's underlying data, should contact Optum Life Sciences, which licenses the data. Free access is provided in the online supplement to the summary statistics from the data, including 16,770 strata and remission rate of various antidepressants in these strata.

## Declaration of Competing Interest

FA, HM, JW, and MY have no conflict of interest to declare. LKB, CAH, and VSN are employees of OptumLabs, a UnitedHealth Group company. LKB, CAH and VSN participated in employee stock purchase program of UnitedHealth Group company.

## Appendix Supplementary materials

Image, application 1

Image, application 1

Image, application 1

## Footnotes

Supplementary material associated with this article can be found in the online version at doi:10.1016/j.eclinm.2021.101171.
