# Supplementary material for: Effectiveness of common antidepressants: a post market release study
Source: eClinicalMedicine. 2021 Oct 25;41:101171. doi: 10.1016/j.eclinm.2021.101171 (PMC8633963; doi:10.1016/j.eclinm.2021.101171)
Supplement: Supplementary file 1 [file mmc1.docx]

**Caption for Supplementary Material**

In the file Subgroups and Optimal Antidepressants, the following information is provided:

| Sheet | Description |
| --- | --- |
| Read Me | Guide to abbreviations and variable names |
| Summ | Summary statistics for the strata for each antidepressant |
| Subgroups and Optimal AD | Strata and the effect size for each antidepressant, each row corresponds to one strata |
| Table 4 | Same Table as Table 4 in the published paper |
| Details | The details of each strata. Several rows correspond to the same strata. Each strata describes the robust, significant, and large predictors of remission or selection bias. |

In the file Subgroups and Optimal Antidepressants, the following information is provided:

| Sheet | Description |
| --- | --- |
| Table 6 | Table 6 in manuscript |
| Count | Distribution of demographics and guide to abbreviations |
| ITT Summ | Performance of models predicting use of specific antidepressants (used as propensity weights for correction of selection bias) |
| ITT Coef | Regression coefficients in predicting use of specific antidepressants. Although all coefficients are presented in one sheet, each antidepressant was predicted by a separate regression |
| Rem Summ | Summary of regressions for predicting remission after taking a specific antidepressant |
| Rem Coef | Coefficients in the regression models used for predicting remission. Although presented in one sheet, each antidepressant was a separate regression. |
| Table 2 | Table 2 in manuscript |
| Table 3 | Table 3 in manuscript, listing a limited number of antidepressants |
| Full Table 3 | Full Table 3 listing all antidepressants |
| Full Table 6 | Full Table 6 listing all antidepressants |
| Table 4 | Table 4 in manuscript, listing a limited number of antidepressants |
| Full Table 4 | Full Table 4, listing all antidepressants examined |
